# Hobotnica: exploring molecular signature quality

**DOI:** 10.12688/f1000research.74846.2

**Published:** 2022-08-16

**Authors:** Alexey Stupnikov, Alexey Sizykh, Anna Budkina, Alexander Favorov, Bahman Afsari, Sarah Wheelan, Luigi Marchionni, Yulia Medvedeva

**Affiliations:** 1 Moscow Institute of Physics and Technology, Moscow, Russian Federation; 2 National Medical Research Center for Endocrinology, Moscow, Russian Federation; 3 Johns Hopkins University, Baltimore, USA; 4 Vavilov Institute for General Genetics RAS, Moscow, Russian Federation; 5 Weill Cornell Medicine, New York, USA; 6 Center of Biotechnology RAS, Moscow, Russian Federation

**Keywords:** Molecular signature, Distance Matrix, Differential Gene Expression, Gene Signature, Rank statistics

## Abstract

A Molecular Features Set (MFS), is a result of a vast diversity of bioinformatics pipelines. The lack of a “gold standard” for most experimental data modalities makes it difficult to provide valid estimation for a particular MFS's quality. Yet, this goal can partially be achieved by analyzing inner-sample Distance Matrices (DM) and their power to distinguish between phenotypes.

The quality of a DM can be assessed by summarizing its power to quantify the differences of inner-phenotype and outer-phenotype distances. This estimation of the DM quality can be construed as a measure of the MFS's quality.

Here we propose Hobotnica, an approach to estimate MFSs quality by their ability to stratify data, and assign them significance scores, that allow for collating various signatures and comparing their quality for contrasting groups.

## Introduction

A signature based on a predefined Molecular Features Set (MFS), which is designed to distinguish biological conditions or phenotypes from each other, is a crucial concept in bioinformatics and precision medicine. In this context, signatures typically originate from MFS from contrasting experimental data from two or more sample groups, which differ phenotypically. These MFS incorporate information on the differences between the groups. The nature of the MFS depends on the modality of the original data. For instance, the MFS provided by the Differential Gene Expression approach is a list of Differentially Expressed genes (DEG); Differential Methylation analysis provides Differentially Methylated Cytosines or regions (DMC and DMR) as MFS.

A significant number of mutational, expression and methylation-based signatures have recently been published and they are actively used in research and translational medicine. Examples of expression-based signatures involve gene sets for clinical prognosis (e.g., PAM50, ^1^ MammaPrint ^2^), for pathways and gene enrichment analysis (e.g., MsigDB collections ^3^), and for drug re-purposing (e.g., LINCS project ^4^).

Direct quality assessment for MFS is currently hardly possible, since there are no ‘gold standard’ datasets where active Molecular Features are explicitly known. In this manuscript, we propose a novel approach - Hobotnica, that allows for measurement of MFS quality by addressing the key property of the signature, namely, its quality for data stratification.

Hobotnica leverages the quality of distance matrices obtained from any source, in order to assess the quality of the MFS from any data modality compared to a random MFS. In this study, we demonstrate its application to transcriptomic and methylation signatures.

## Methods

### Approach

The Hobotnica approach is as follows: For a given data set W and a given MFS ( S ) we derive the inter-sample distance matrix ( $\mathrm{DM}\left(S,W\right)$ ). Then we assess the quality of DM (and, thus, of S ) with a summarizing function ( $\alpha \left(\mathrm{DM}\left(S\right)\right)=\alpha \left(\mathrm{DM}\left(S\right),Y\right)$ or by abuse of notation $\alpha \left(\mathrm{DM}\left(S\right)\right)$ ) where ( Y ) represents the labels of samples. In shorter notation,

$$\begin{array}{c} H:S\\ f\left(S|D\right)\to \mathrm{DM}\\ g\left(\mathrm{DM}|Y\right)\to \alpha \end{array}$$ (1)

We desire the function α to gauge if the inner-class samples are closer to each other than to outer-class samples. If no difference exists from one class to another, α must be close to zero and as the difference grows, α grows. In the ideal case of a perfect separation, α reaches its maximum at 1:

•
$\alpha \in \left[0,1\right]$

•
α→1⇔ High groups stratification quality
•
α→0⇔ Low groups stratification quality

Under the null hypothesis of Hobotnica (( $H_{0}$ )), no significant difference exists between $\alpha \left(S\right)$ and the α of an equal-sized general random set. On the contrary, the alternative ( $H_{A}$ ) hypothesizes that S generates higher α than most random $S^{\prime }$ of the same size. To estimate a null distribution for Hobotnica’s α , we applied a permutation test. As our default options, we use Kendall distance as the distance measure and Mann-Whitney-Wilcoxon test as the summarizing function.

When instead of a single MFS a set of hypotheses $\left\{H_{1}:{\mathrm{MFS}}_{1},H_{2}:{\mathrm{MFS}}_{2},\ldots ,H_{n}:{\mathrm{MFS}}_{n}\right\}$ is in place, for each Molecular Feature Set ${\mathrm{MFS}}_{i}$ corresponding Distance Matrix ${\mathrm{DM}}_{i}$ can be generated, and than, in turn, particular value of the measure $\alpha_{i}$ :

$$\left\{\begin{array}{l} H_{1}:{\mathrm{MFS}}_{1}\\ H_{2}:{\mathrm{MFS}}_{2}\\ \ldots \\ H_{n}:{\mathrm{MFS}}_{n} \end{array}\right.\to \left\{\begin{array}{l} f\left({\mathrm{MFS}}_{1}|D\right)\to {\mathrm{DM}}_{1}\\ f\left({\mathrm{MFS}}_{2}|D\right)\to {\mathrm{DM}}_{2}\\ \ldots \\ \left({\mathrm{MFS}}_{n}|D\right)\to {\mathrm{DM}}_{n} \end{array}\right.\to \left\{\begin{array}{l} g\left({\mathrm{DM}}_{1}|A\right)\to \alpha_{1}\\ g\left({\mathrm{DM}}_{2}|A\right)\to \alpha_{2}\\ \ldots \\ g\left({\mathrm{DM}}_{n}|A\right)\to \alpha_{n} \end{array}\right..$$ (2)

Thus, for every MFS ${\mathrm{MFS}}_{i}$ from set of hypotheses $\left\{H_{1}:{\mathrm{MFS}}_{1},H_{2}:{\mathrm{MFS}}_{2},\ldots ,H_{n}:{\mathrm{MFS}}_{n}\right\}$ H-score $\alpha_{i}$ may be computed, resulting in a set $\left\langle \alpha_{1},\alpha_{2},\ldots \alpha_{n},\right\rangle$ . Comparing α values allows for corresponding Feature Sets qualities ranking and selecting the most informative Signatures for the Data D .

### Model

**Figure 1.**
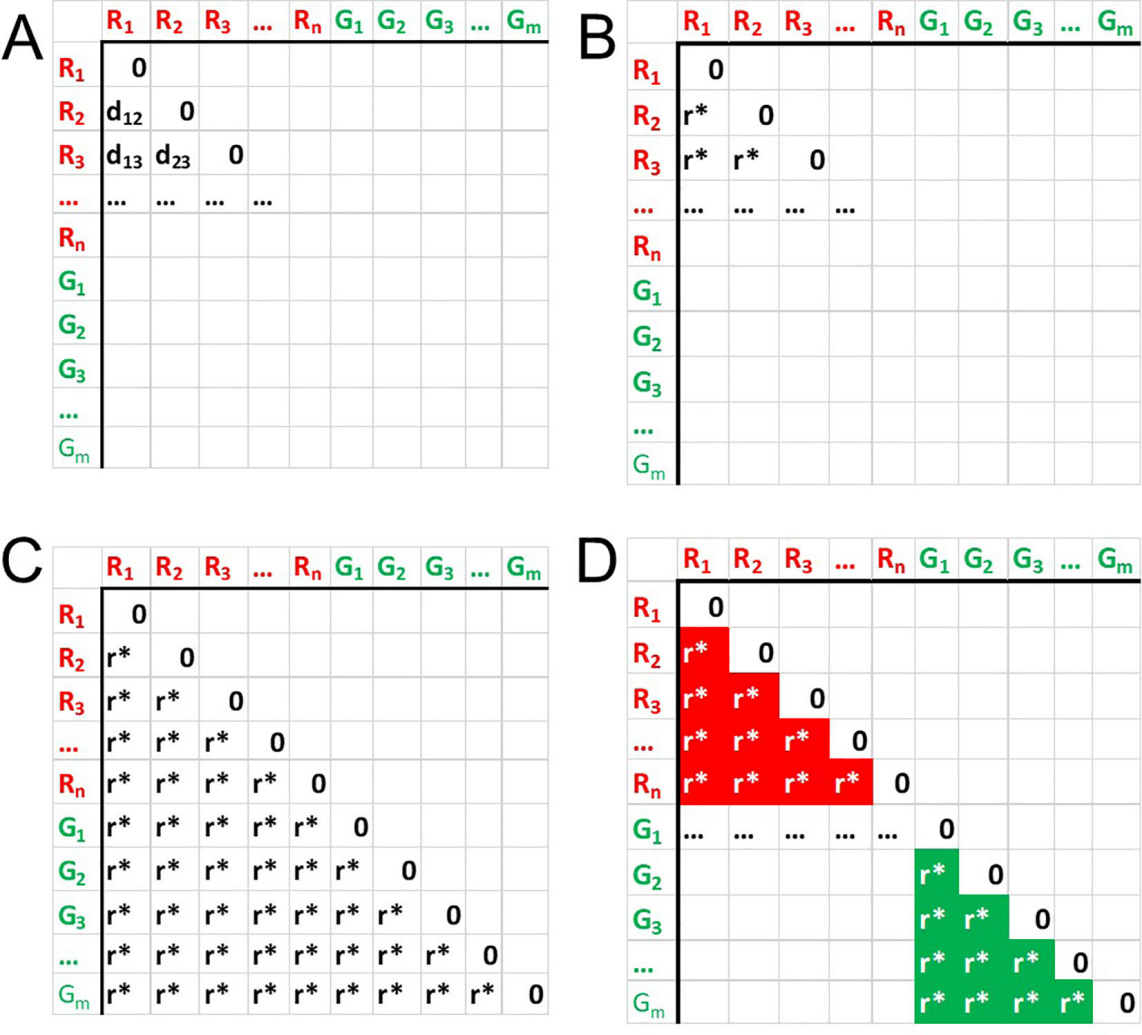
Distances ranking procedure A: Distance matrix structure. B: each distance element *d*_*pq*_ is substituted with corresponding rank value. C: Repeating the process for all elements of the distance matrix. D: Sub-setting in-class ranks corresponding to in-class distance elements.

For two groups of samples, *R* and *G*, taking into account the nature of Distance Matrix (0-diagonal and symmetry), the corresponding lower triangle matrix may be considered ( Figure 1A). The distance values of this matrix *d*_*pq*_ can be ranked ( Figure 1B), producing new matrix with natural values *r*_*pq*_ ( Figure 1C).

Subseting the matrix to values corresponding to in-class distances ( Figure 1D), we can compute sum of these ranks Σ.

Given the numbers of samples is *n* in group 1 and in *m* group 2, total number of ranks (and therefore max value of a rank) in un-subsetted triangle matrix will be

$$M=\frac{\left(m+n)(m+n-1\right)}{2}$$ (3)

Likewise, total number of ranks is subsetted to in-class values will be

$$N=\frac{\left(m^{2}+n^{2}-(m+n)\right)}{2}$$ (4)

Σ reaches its min value *A* if the subset procedure selects minimal values of ranks, or, in other words, ranks in the selected part of rank matrix are values from 1 to *M*:

$$A=\sum_{i=1}^{M}i$$ (5)

The minimal value is reached when the lowest ranks reside in the diagonal squares of the distance matrix that correspond to in-group distances, and, therefore, best groups separation. Similarly, max value *B* is delivered when maximal values of Ranks Matrix are selected - from ( *N* − *M*) to *N*:

$$B=\sum_{i=(N-M)}^{N}i$$ (6)

Thus, Σ ∈ [ *A*, *B*]. Now performing a scaling of compact [ *A*, *B*] to [0, 1] finally allows us to retrieve value *α* with requested properties:

F:[A,B]→[0,1],F(A)=1,F(B)=0. (7)

F:S→α (8)

This procedure finally allows to introduce measure *α* with necessary listed properties. We refer to this measure as *Hobotnica*, or H-score.

Thus, for every Gene Signature *GS*_*i*_ from set of hypotheses { *H*_1_ : *GS*_1_, *H*_2_ : *GS*_2_, ..., *H*_*n*_ : *GS*_*n*_} H-score *α*_*i*_ may be computed, resulting in a set 〈 *α*_1_, *α*_2_, ... *α*_*n*_〉. Comparing and ranking *α* values allows for corresponding Gene Signatures qualities ranking and comparison.

The proposed approach is different from existing metrics, such as often used Rand index ^5^ and other clusteringbased measures, as it allows one to avoid clustering procedure, that itself may be carried out with various approaches and parameters. ^6^^–^^8^ In contract to clustering-based methods, H-scores directly reflects the sample stratification quality.

### Validation

To validate our approach, we conducted four case studies.

In the first case study we extracted RNA-seq expression dataset for prostate cancer from the Cancer Genome Atlas ( TCGA) on counts level. ^9^ As MFSs, we recruited the C2 collection of molecular signatures from MSigDB, ^3^^,^^10^ that contains a number of prostate-related gene sets. This way, every candidate MFS (gene set from the collection) produced its specific H-score.

For the second case study, we recruited the PAM50 molecular signature, ^11^ which was designed for classifying various breast cancer subtypes, as MFS. Then, we applied it to several datasets containing these breast cancer subtypes. ^9^^,^^12^^–^^15^

In the third case study, we explored H-scores delivered by various DGE approaches. We performed DGE analysis for two groups of mice samples with different response to MYC factor treatment (Mycfl/fl vs Myc Δ IE, ERT2 genotypes) ^16^ with DESeq2 ^17^ and edgeR. ^18^

The top 100 genes for each method were then retrieved. In addition, we extracted a list of genes genes with the highest variance in expression, as well as a number of random gene sets.

In each case, the counts were normalised to counts per million (cpm). For every geneset an H-score and its p-value with BH ^19^ correction were computed.

In the last case the Hobotnica application for differential methylation signatures assessment was demonstrated. Hobotnica was applied to the signature from study ^20^ that distinguish B-cell subpopulations with mutated and unmutated IGHV from patients with chronic lymphocytic leukemia (M-CLL and U-CLL). The signature was derived from the data obtained on 450k Human Methylation Array, the length of the signature is 3265 sites.

The signature was validated on datasets from other experiments containing the same comparison groups (M-CLL and U-CLL): GSE136724 from, ^21^ GSE143411 from ^22^ and GSE144894 from. ^23^ Datasets GSE136724 and GSE143411 contain samples from patients after chemo(immune) therapy and untreated samples, only untreated groups were used for validation. H-score was calculated for each dataset using matrices of beta values with beta-mixture quantile normalization. The signature was reduced to 3089 sites for GSE136724, 3091 sites for GSE143411 and 3254 for GSE144894. The p-value was calculated based on 100000 random signatures of the same length using pseudocount.

## Results

**Figure 2.**
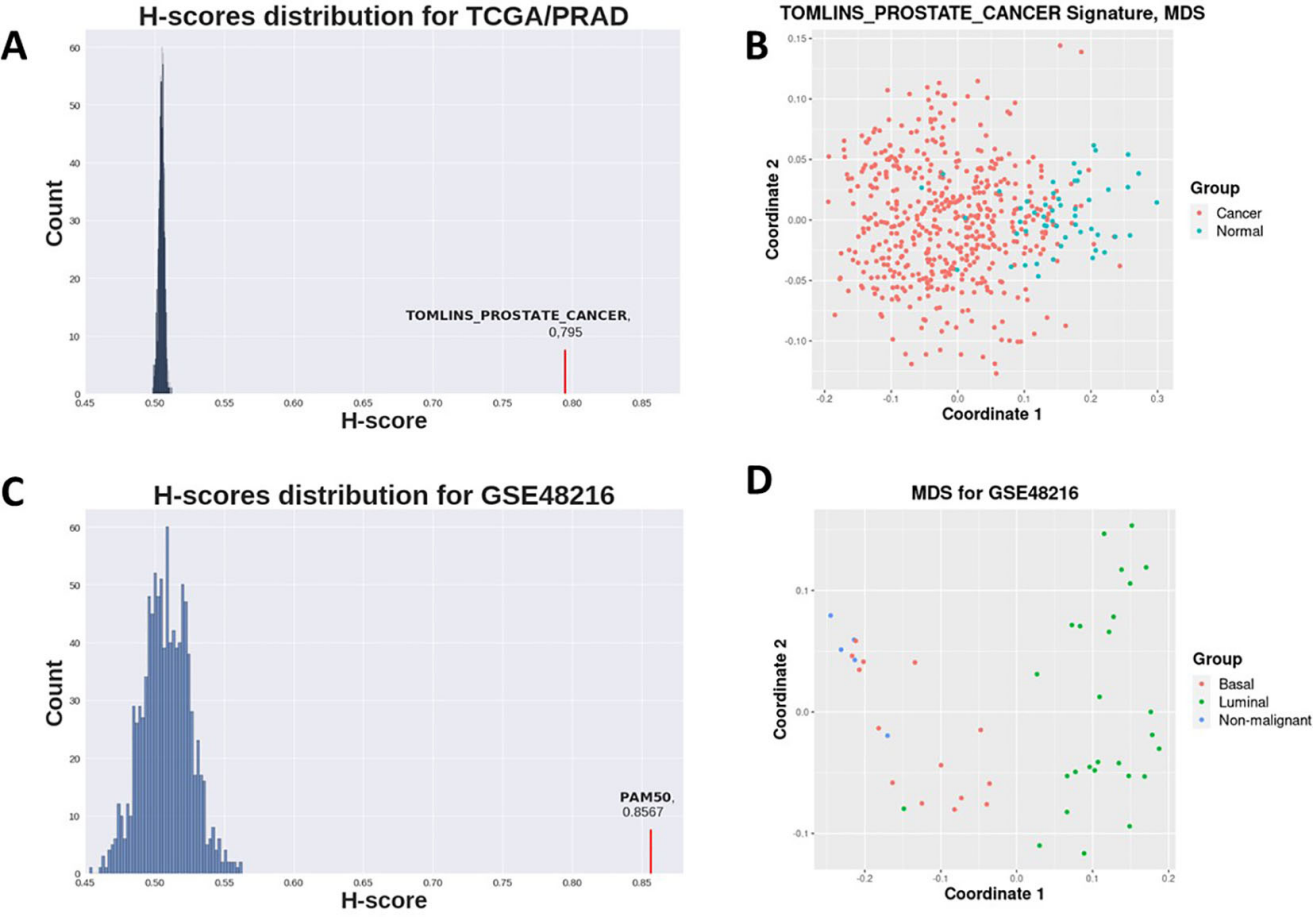
A : Distribution of H-scores for random genesets (blue) on TCGA prostate cancer vs normal dataset (see Table 1) and Tomlins prostate geneset H-score (red). B: MDS for TCGA prostate demonstrates samples separation with Tomlins geneset. C: Distribution of H-scores for random genesets (blue) on GSE48216 breast cancer dataset (see Table 2) and PAM50 geneset H-score (red). D: MDS for GSE48216 breast cancer dataset samples separation with PAM50 geneset.

Prostate-related C2 gene sets clearly demonstrated highest H-score values and sufficient statistical significance ( Table 1, Figure 2A), as well as data stratification ( Figure 2B), which is expected for prostate cancer as opposed to control contrast. Gene sets not attributed to prostate cancer-related processes did not achieve statistically significant p-values ( Table 1).

**Table 1.** Ten C2-chemical and genetic perturbations (GCP) Gene Signatures with the highest H-scores.

Signature	H-score	p-value
**TOMLINS_PROSTATE_CANCER**	0.795	**0.025**
**WALLACE_PROSTATE_CANCER**	0.747	**0.025**
**OUYANG_PROSTATE_CANCER_PROGRESSION**	0.745	**0.025**
**LIU_PROSTATE_CANCER**	0.735	**0.025**
PIEPOLI_LGI1_TARGETS	0.724	0.059
SMID_BREAST_CANCER_RELAPSE_IN_LIVER	0.712	0.164
TIMOFEEVA_GROWTH_STRESS_VIA_STAT1	0.708	0.240
GENTILE_UV_LOW_DOSE	0.705	0.308
JOHANSSON_BRAIN_CANCER_EARLY_VS_LATE	0.701	0.377
HOWLIN_CITED1_TARGETS_1	0.700	0.377

**Figure 3.**
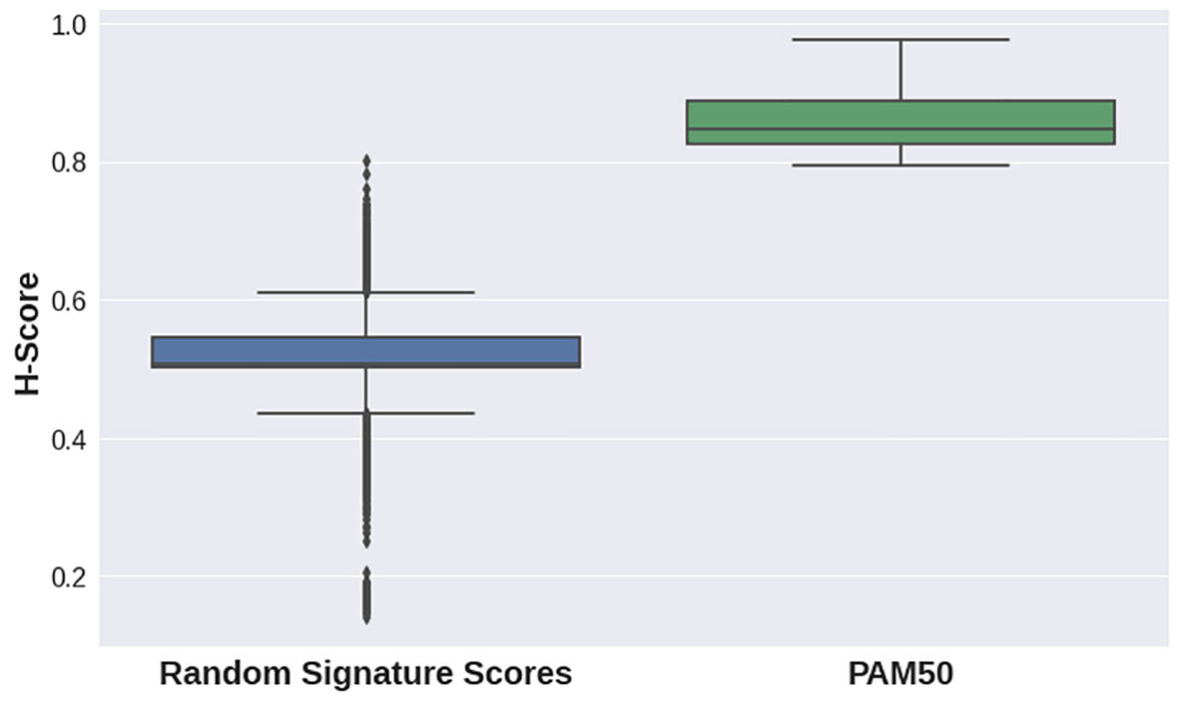
Distribution of random gene sets-delivered (blue) and PAM50 gene set-delivered (green) H-scores for breast cancer datasets (see Table 2).

H-scores for the PAM50 signature were evidently higher for all datasets in the second case study than those for random gene sets for the same datasets ( Figure 3, Figure 2C). This implies that the PAM50 signature exhibits a high stratification quality for various breast cancer subtypes samples. PAM50-delivered H-scores also demonstrated highly statistically significant p-values ( Table 2).

**Table 2.** PAM50 results.

GEO Accession	Sample size	Groups in dataset	H-score	p-value
GSE58135	168	6	0.772	7e-4
GSE62944	1067	5	0.8892	0.0003
GSE48216	46	3	0.8567	0.0003
GSE80333	10	3	0.9765	0.0003

**Figure 4.**
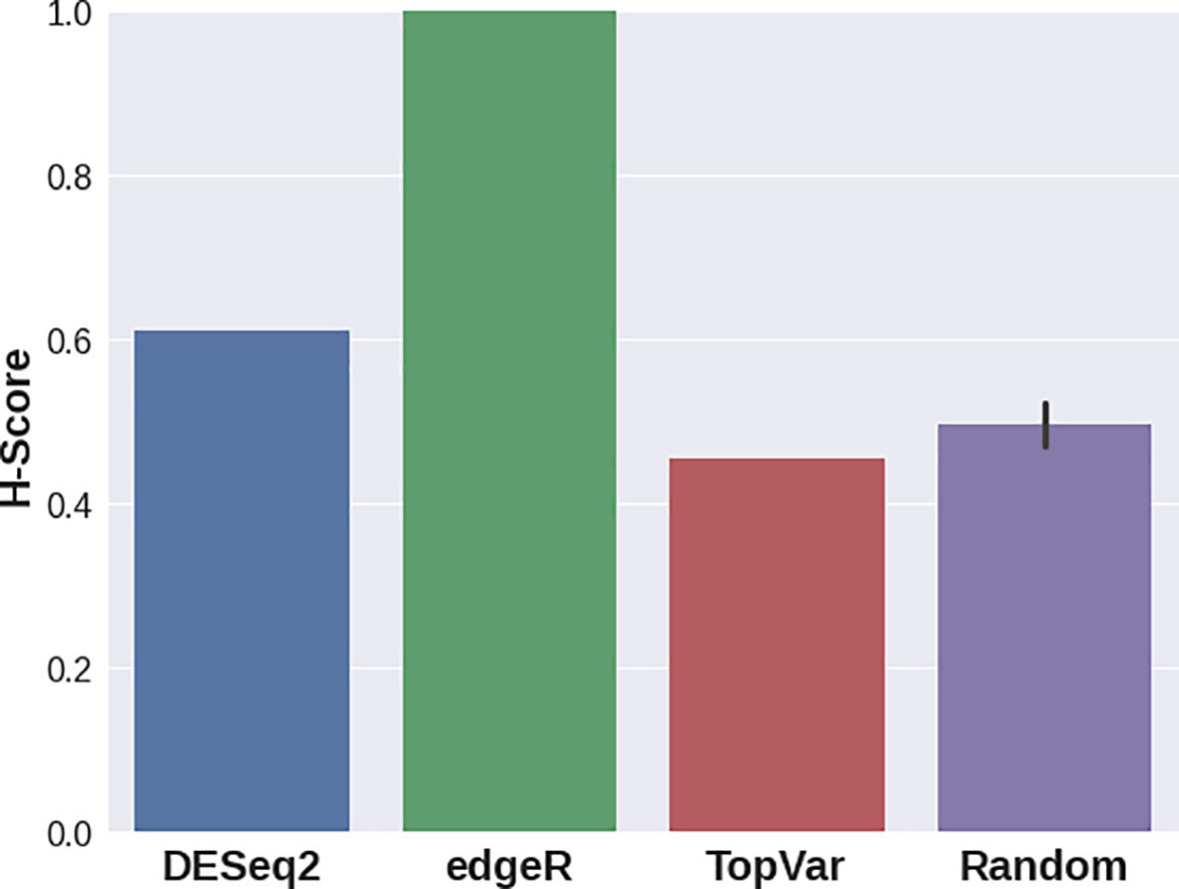
H-scores for the top 100 Gene Signatures delivered from DESeq2, edgeR, genes with highes variance and random gene sets applied to GSE155460 data.

In the third case study, various DGE approaches resulted in gene sets that delivered significantly different H-scores ( Figure 4). For this dataset, edgeR provided a signature with the best quality score, while DESeq2 still demonstrated a higher separation quality than that of random signatures. Genes with the highest variance showed lower scores compared to random gene sets. This result stresses the importance of the Hobotnica procedure to evaluate the quality of a particular DGE analysis.

**Figure 5.**
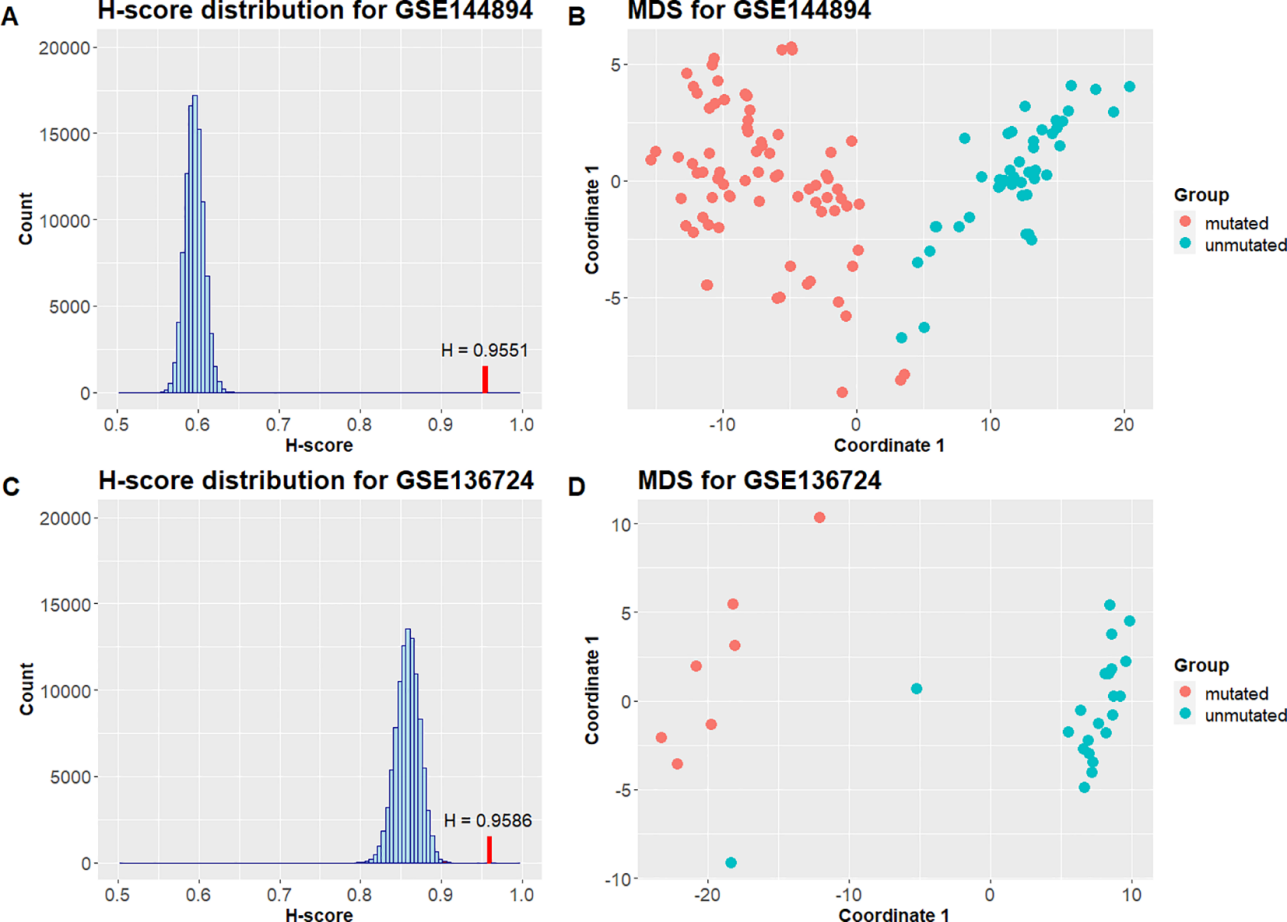
A: H-score distribution for GSE144894 dataset (blue) and H-score corresponding to the validated signature (red) B: MDS plot for GSE144894 samples based on the differential methylation signature C: H-score distribution for GSE136724 dataset (blue) and H-score corresponding to the validated signature (red) B: MDS plot for GSE136724 samples from untreated group based on the differential methylation signature.

Table 3 contains the result H-scores and the corresponding p-values for differential methylation signature validation. H-score values are close to 1, and the p-values are less than 0.05 for all tested datasets. The distributions of Hscores obtained from 100000 random signatures of the same length that were used for p-value calculation are shown in Figure 5. Figure 5 displays MDS plots based on submatrices that include only differential methylation signature sites.

**Table 3.** H-scores and p-values for the differential methylation signature that differentiate M-CLL and UCLL.

GEO Accession	Unmutated sample size	Mutated sample size	H-score	p-value
GSE136724	22	7	0.9586	9.9999e-06
GSE143411	8	2	1	9.9999e-06
GSE144894	44	76	0.9551	9.9999e-06

## Discussion

Hobotnica was designed to quantitatively evaluate MFS quality through their ability for data stratification, based on their inter-sample distance matrices, and to assess the statistical significance of the results. We demonstrated that Hobotnica can efficiently estimate the quality of a molecular signature in the context of expression data.

The suggested method can be used to evaluate various sorts of MFSs: those retrieved from DGE or DM analyses, Mutation/single nucleotide variation calling or pathways analysis, as well as data modalities of other types, that are suitable as differential problems.

The non-parametric statistic used in the approach not only allows for MFS of various types (differential, predefined, etc.) and data modalities (expression, methylation, etc.), but also for different structure of contrasted samples groups (sample size, preprocessing methods, etc.).

A possible application of Hobotnica is evaluating a particular model’s performance (e.g., DGE model) for a particular dataset. This will allow researchers to choose a method that delivers a signature with the best data stratification from a number of proposed approaches.

Assessing H-score values for various lengths of the same set or signature can be explored with the proposed method, which will help to optimize MFS structure. Such procedures can be especially crucial in clinical applications.

## Data availability

### Underlying data

NCBI Gene Expression Omnibus: Alternatively processed and compiled RNA-Sequencing and clinical data for thousands of samples from The Cancer Genome Atlas, https://identifiers.org/ncbiprotein:GSE62944

NCBI Gene Expression Omnibus: Modeling precision treatment of breast cancer, https://identifiers.org/ncbiprotein:GSE48216

NCBI Gene Expression Omnibus: Spatial proximity to fibroblasts impacts molecular features and therapeutic sensitivity of breast cancer cells influencing clinical outcomes, https://identifiers.org/ncbiprotein:GSE80333

NCBI Gene Expression Omnibus: Next Generation Sequencing Analysis of Mycfl/fl and MycIE, ERT2 intestinal transcriptomes, https://identifiers.org/ncbiprotein:GSE155460

NCBI Gene Expression Omnibus: DNA methylation of chronic lymphocytic leukemia with differential response to chemotherapy, https://identifiers.org/ncbiprotein:GSE136724

NCBI Gene Expression Omnibus: A dataset of sequential DNA methylation profiles (2 timepoints) of 10 patients with chronic lymphocytic leukemia, https://identifiers.org/ncbiprotein:GSE143411

NCBI Gene Expression Omnibus: CLL intraclonal fractions exhibit established and recently-acquired patterns of DNA methylation [ME], https://identifiers.org/ncbiprotein:GSE144894

### Extended data

Analysis code

Analysis code available from: https://github.com/lab-medvedeva/Hobotnica-main

Archived analysis code as at time of publication: https://doi.org/10.5281/zenodo.5656814

License: GNU General Public License v2.0

## References

[1] Parker JS Mullins M Cheang MCU : Supervised risk predictor of breast cancer based on intrinsic subtypes. *J. Clin. Oncol.* 2009;27(8):1160–1167. 10.1200/JCO.2008.18.1370 19204204 PMC2667820

[2] Cardoso F van’t Veer LJ Bogaerts J : 70-gene signature as an aid to treatment decisions in earlystage breast cancer. *N. Engl. J. Med.* 2016;375(8):717–729. 10.1056/NEJMoa1602253 27557300

[3] Subramanian A Tamayo P Mootha VK : Gene set enrichment analysis: a knowledge-based approach for interpreting genome-wide expression profiles. *Proc. Natl. Acad. Sci.* 2005;102(43):15545–15550. 10.1073/pnas.0506580102 16199517 PMC1239896

[4] Liu C Jing S Yang F : Compound signature detection on lincs l1000 big data. *Mol. BioSyst.* 2015;11(3):714–722. 10.1039/C4MB00677A 25609570 PMC4333019

[5] Rand WM : Objective criteria for the evaluation of clustering methods. *J. Am. Stat. Assoc.* 1971;66(336):846–850.

[6] Abbas OA : Comparisons between data clustering algorithms. *Int. Arab. J. Inf. Technol.* 2008;5(3).

[7] Chen G Jaradat SA Banerjee N : Evaluation and comparison of clustering algorithms in analyzing es cell gene expression data. *Stat. Sin.* 2002;241–262.

[8] Kafieh R Mehridehnavi A : A comprehensive comparison of different clustering methods for reliability analysis of microarray data. *J. Med. Signals Sens.* 2013;3(1):22. 24083134 PMC3785067

[9] Rahman M Jackson LK Evan Johnson W : Alternative preprocessing of rna-sequencing data in the cancer genome atlas leads to improved analysis results. *Bioinformatics.* 2015;31(22):3666–3672. 10.1093/bioinformatics/btv377 26209429 PMC4804769

[10] Liberzon A Subramanian A Pinchback R : Molecular signatures database (MSigDB) 3.0. *Bioinformatics.* 05 2011;27(12):1739–1740. ISSN 1367-4803. 10.1093/bioinformatics/btr260 21546393 PMC3106198

[11] Parker JS Mullins M Cheang MCU : Supervised risk predictor of breast cancer based on intrinsic subtypes. *J. Clin. Oncol.* 2009;27(8):1160–1167. 10.1200/JCO.2008.18.1370 19204204 PMC2667820

[12] Varley KE Gertz J Roberts BS : Recurrent read-through fusion transcripts in breast cancer. *Breast Cancer Res. Treat.* 2014;146(2):287–297. 10.1007/s10549-014-3019-2 24929677 PMC4085473

[13] Marusyk A Tabassum DP Janiszewska M : Spatial proximity to fibroblasts impacts molecular features and therapeutic sensitivity of breast cancer cells influencing clinical outcomes. *Cancer Res.* 2016;76(22):6495–6506. 10.1158/0008-5472.CAN-16-1457 27671678 PMC5344673

[14] Daemen A Griffith OL Heiser LM : Modeling precision treatment of breast cancer. *Genome Biol.* 2013;14(10):R110–R114. 10.1186/gb-2013-14-10-r110 24176112 PMC3937590

[15] Costello JC Heiser LM Georgii E : A community effort to assess and improve drug sensitivity prediction algorithms. *Nat. Biotechnol.* 2014;32(12):1202–1212. 10.1038/nbt.2877 24880487 PMC4547623

[16] Luo Y Yang S Wu X : Intestinal MYC modulates obesity-related metabolic dysfunction. *Nat. Metab.* July 2021;3(7):923–939. 10.1038/s42255-021-00421-8 34211180 PMC9944847

[17] Love MI Huber W Anders S : Moderated estimation of fold change and dispersion for rna-seq data with deseq2. *Genome Biol.* 2014;15(12):1–21. 10.1186/s13059-014-0550-8 PMC4302049 25516281

[18] Robinson MD McCarthy DJ Smyth GK : edger: a bioconductor package for differential expression analysis of digital gene expression data. *Bioinformatics.* 2010;26(1):139–140. 10.1093/bioinformatics/btp616 19910308 PMC2796818

[19] Benjamini Y Hochberg Y : Controlling the false discovery rate: a practical and powerful approach to multiple testing. *J. R. Stat. Soc. Series B* 1995;57(1):289–300.

[20] Kulis M Heath S Bibikova M : Epigenomic analysis detects widespread gene-body DNA hypomethylation in chronic lymphocytic leukemia. *Nat. Genet.* Nov 2012;44(11):1236–1242. 23064414 10.1038/ng.2443

[21] Yosifov DY Bloehdorn J Döhner H : DNA methylation of chronic lymphocytic leukemia with differential response to chemotherapy. *Sci. Data.* 05 2020;7(1):133. 32358561 10.1038/s41597-020-0456-0 PMC7195470

[22] Zapatka M Tausch E Öztürk LM : Clonal evolution in chronic lymphocytic leukemia is scant in relapsed but accelerated in refractory cases after chemo(immune) therapy. *Haematologica.* 03 2022;107(3):604–614. 10.1186/gb-2013-14-10-r110 33691380 PMC8883533

[23] Bartholdy BA Wang X Yan XJ : CLL intraclonal fractions exhibit established and recently acquired patterns of DNA methylation. *Blood Adv.* 03 2020;4(5):893–905. 10.1186/gb-2013-14-10-r110 32150608 PMC7065474

