# Supplementary material for: Hobotnica: exploring molecular signature quality
Source: F1000Res. 2022 Aug 16;10:1260. Originally published 2021 Dec 8. [Version 2] doi: 10.12688/f1000research.74846.2 (PMC9513410; doi:10.12688/f1000research.74846.2)
Supplement: Peer review history

# Peer review: Reviewer response for version 2

**DOI:** 10.5256/f1000research.134369.r147651

**Published:** 2022-09-26

**Authors:** Roberto Malinverni

**Affiliations:** 1 Cancer and Leukemia Epigenetics and Biology Program, Josep Carreras Leukemia Research Institute (IJC), Badalona, Spain

In my opinion the authors answer to all my criticism. In this version of the article, they correct some imprecision in the graphs and add figures that help to understand the method. I think that now is ready for indexing.

Is the rationale for developing the new method (or application) clearly explained?

Yes

Is the description of the method technically sound?

Partly

Are the conclusions about the method and its performance adequately supported by the findings presented in the article?

Partly

If any results are presented, are all the source data underlying the results available to ensure full reproducibility?

Yes

Are sufficient details provided to allow replication of the method development and its use by others?

Partly

Reviewer Expertise:

Epigenetics. R-developrer

I confirm that I have read this submission and believe that I have an appropriate level of expertise to confirm that it is of an acceptable scientific standard.

# Peer review: Reviewer response for version 2

**DOI:** 10.5256/f1000research.134369.r147650

**Published:** 2022-09-20

**Authors:** Shailesh Tripathi

**Affiliations:** 1 Production and Operations Management, University of Applied Sciences Upper Austria, Linz, Austria; 2 FH Austria, Steyr, Austria

I have gone through the responses provided by the authors and the updates of the paper. The author has addressed the main questions and revised the manuscript. I have no further questions. For the final acceptance, I approve it.

Is the rationale for developing the new method (or application) clearly explained?

Partly

Is the description of the method technically sound?

Partly

Are the conclusions about the method and its performance adequately supported by the findings presented in the article?

Partly

If any results are presented, are all the source data underlying the results available to ensure full reproducibility?

Yes

Are sufficient details provided to allow replication of the method development and its use by others?

Yes

Reviewer Expertise:

Data science, Machine learning, network analysis, computational biology, gene expression data analysis

I confirm that I have read this submission and believe that I have an appropriate level of expertise to confirm that it is of an acceptable scientific standard.

# Peer review: Reviewer response for version 1

**DOI:** 10.5256/f1000research.78645.r102284

**Published:** 2022-01-05

**Authors:** Shailesh Tripathi

**Affiliations:** 1 Production and Operations Management, University of Applied Sciences Upper Austria, Linz, Austria; 2 FH Austria, Steyr, Austria

The authors present an approach called Hobotonica for quantitatively evaluating (by assigning H score) MFS quality for given sample labels. This approach could be useful for analyzing samples, for e.g., quality comparison, filtering out poor quality samples, and comparing different phenotypical conditions and experiments. It is important that the authors should discuss a reasonable H-score interpretation in terms of various implications of data quality/outcome related to experimental conditions, sample size, data preprocessing, and the complexity of biological systems reflecting the non-trivial correlation structure.

 I highlight some of the recommendations to be discussed in the paper:

1. The author should add simulation studies providing a realistic understanding and interpretation of the H score.
2. How is the current approach different from the clustering-based approach where the optimized number of clusters are compared to sample labels using rand-index (where a high rand score means the clustering solution and the sample labels are in agreement) or other measures.
3. The analysis should consider experimental conditions (data derived from multiple experiments representing the same phenotype), data preprocessing methods, sample size, and gene expression data covariance structure.
4. How does H-score vary with relation to the number of phenotype conditions and number of MFS. The authors should add analysis and interpretation of results:
  - when MFS is differentially expressed genes.
  - when MFS is randomly selected.
  - When MFS is a predefined set (e.g., GO pathway).
5. The author should add accurate descriptions of all the notations used.
6. Add a definition of H-score.

Is the rationale for developing the new method (or application) clearly explained?

Partly

Is the description of the method technically sound?

Partly

Are the conclusions about the method and its performance adequately supported by the findings presented in the article?

Partly

If any results are presented, are all the source data underlying the results available to ensure full reproducibility?

Yes

Are sufficient details provided to allow replication of the method development and its use by others?

Yes

Reviewer Expertise:

Data science, Machine learning, network analysis, computational biology, gene expression data analysis

I confirm that I have read this submission and believe that I have an appropriate level of expertise to confirm that it is of an acceptable scientific standard, however I have significant reservations, as outlined above.

# Response

**Published:** 2022-05-23

**Authors:** Alexey Stupnikov

**Affiliations:** Moscow Institute of Physics and Technology, Russian Federation

- **The author should add simulation studies providing a realistic understanding and interpretation of the H score.**

​​​​​​​The Reviewer raises an important problem of parametric and nonparametric statistics. The nature of H-score distribution indeed was not discussed in detail. Yet, its distribution was not the focus of this study, for distribution of H-scores for randomly selected Molecular Feature Sets is only employed to compute empirical p-values. This procedure is nonparametric and, therefore, is not affected by the nature of H-score distribution. We agree the nonparametric nature of the statistic needs to be mentioned more explicitly in the manuscript, and add a paragraph to the Discussion section:

 “ *The non-parametric statistic used in the approach not only allows for MFS of various types (differential, predefined, etc.) and data modalities (expression, methylation, etc.), but also for different structure of contrasted samples groups (sample size, preprocessing methods, etc.)*”

- **How is the current approach different from the clustering-based approach where the optimized number of clusters are compared to sample labels using rand-index (where a high rand score means the clustering solution and the sample labels are in agreement) or other measures.**

We agree the difference of our approach from clustering-based methods was not mentioned explicitly. We have added a paragraph to the Methods section discussing the difference of our approach.

****

*“The proposed approach is different from existing metrics, such as often used Rand index and other clustering-based measures, as it allows one to avoid clustering procedure, that itself may be carried out with various approaches and parameters. In contract to clustering-based methods, H-scores directly reflects the sample stratification quality.”*

- **The analysis should consider experimental conditions (data derived from multiple experiments representing the same phenotype), data preprocessing methods, sample size, and gene expression data covariance structure.**

The dataset details the reviewer mentions are crucial for understanding a particular dataset’s structure, and it is correct that they may affect some types of analysis. However, since the nature of the statistic is nonparametric, the details do not affect our approach. We agree this point needs to be stressed explicitly in the manuscript, and add a paragraph in the Discussion section:

 “ *The non-parametric statistic used in the approach not only allows for MFS of various types (differential, predefined, etc.) and data modalities (expression, methylation, etc.), but also for different structure of contrasted samples groups (sample size, preprocessing methods, etc.)*”

 The exploration of H-score distribution in relation to experimental conditions mentioned above certainly is an interesting fundamental question. However, it does not affect the practical implementation we introduce. Therefore, we believe that such study is beyond the scope of a current paper.

  

- **How does H-score vary with relation to the number of phenotype conditions and number of MFS. The authors should add analysis and interpretation of results:**
  - **when MFS is differentially expressed genes.**
  - **when MFS is randomly selected.**
  - **When MFS is a predefined set (e.g., GO pathway). **

The nature of MFS indeed may be quite different. For this reason we have performed and discussed the analysis of following MFS types:

- differentially expressed genes
- randomly selected genes
- MysigDB gene sets (predefined genesets)

In the revised version of manuscript differentially methylated MFS are also considered.

 In this way, we feel the analysis and the interpretation the Reviewer suggested can be found in the manuscript.

  

- **The author should add accurate descriptions of all the notations used.**

To address Reviewer’s comment we carefully checked the manuscript to ensure all introduced notations are defined and explained, and added a subsection to the Methods section for more detailed description of H-score.

  

- **Add a definition of H-score.**

We agree the definition of the H-score statistic and notation we introduce should be expanded. To address Reviewer’s comment, we have added a subsection to the Methods section where we define H-score with more details.

# Peer review: Reviewer response for version 1

**DOI:** 10.5256/f1000research.78645.r102280

**Published:** 2021-12-20

**Authors:** Roberto Malinverni

**Affiliations:** 1 Cancer and Leukemia Epigenetics and Biology Program, Josep Carreras Leukemia Research Institute (IJC), Badalona, Spain

In this short article the authors present an R package called Hobotnica, whose purpose is to evaluate the goodness with which different methodologies can stratify the results presented as Molecular Feature Sets (MFS). With MFS the authors point to all those types of data as a result of different - *omics* techniques (such as expression, methylation, Mutation / single nucleotide variation calling or pathways analysis). 

**Major comments**

1. The authors present three examples in which it is demonstrated how this approach is able to evaluate the effectiveness of MFS stratification, but the examples considered are all based on expression data. To verify the statements presented in the article, it would be useful to test the methodology on different data (for example methylation arrays). The approach chosen for this evaluation is based on the calculation and comparison of Distance Matrices (DM). 
2. The example of figure 3 evaluates two different standard approaches for the analysis of RNAseq using Hobotnica and the H0 value as discriminant. It can be appreciated in this figure how the stratification quality of Deseq2 is decidedly more efficient than both random genes and top variant genes. Surprisingly, however, the H0 value calculated using the top 100 genes collected with edgeR is very similar to that calculated using random genes, this confused me. Authors should explain this similarity more in depth. 

 

 The data presented in this article do not seem to convince satisfactorily. The quality evaluation power obtained by applying Hobotnica does not seem to correspond to the premises made. While not in fact a slate on the methodology, my advice is to review the examples and try to improve in benchmarking, adding different types of data.

Is the rationale for developing the new method (or application) clearly explained?

Yes

Is the description of the method technically sound?

Partly

Are the conclusions about the method and its performance adequately supported by the findings presented in the article?

Partly

If any results are presented, are all the source data underlying the results available to ensure full reproducibility?

Yes

Are sufficient details provided to allow replication of the method development and its use by others?

Partly

Reviewer Expertise:

Epigenetics. R-developrer

I confirm that I have read this submission and believe that I have an appropriate level of expertise to confirm that it is of an acceptable scientific standard, however I have significant reservations, as outlined above.

# Response

**Published:** 2022-05-23

**Authors:** Alexey Stupnikov

**Affiliations:** Moscow Institute of Physics and Technology, Russian Federation

- **The authors present three examples in which it is demonstrated how this approach is able to evaluate the effectiveness of MFS stratification, but the examples considered are all based on expression data. To verify the statements presented in the article, it would be useful to test the methodology on different data (for example methylation arrays). The approach chosen for this evaluation is based on the calculation and comparison of Distance Matrices (DM).**

We thank the Reviewer for this suggestion. Indeed, the method we propose is applicable for Molecular Feature Sets evaluation of different nature, yet in the manuscript we demonstrated its work only for Expression based data.To improve the manuscript in a way the Reviewer suggested , we have performed additional analysis on Methylation based data for several datasets. We added this case study to the Validation and Results sections and comments to the Discussion section.


** **

- **The example of figure 3 evaluates two different standard approaches for the analysis of RNAseq using Hobotnica and the H0 value as discriminant. It can be appreciated in this figure how the stratification quality of Deseq2 is decidedly more efficient than both random genes and top variant genes. Surprisingly, however, the H0 value calculated using the top 100 genes collected with edgeR is very similar to that calculated using random genes, this confused me. Authors should explain this similarity more in depth. **

** **


 The point the Reviewer mentions indeed raises concern. After thorough examination we identified a data analysis related problem that caused the wrong genes subset process and resulted in the incorrect depiction in the presented barplot. We have corrected this issue. The rest of our findings were not changed during our reevaluation and all the results hold. The results are depicted and Fig.5 in the current manuscript version. 

  

- **The data presented in this article do not seem to convince satisfactorily. The quality evaluation power obtained by applying Hobotnica does not seem to correspond to the premises made. While not in fact a slate on the methodology, my advice is to review the examples and try to improve in benchmarking, adding different types of data.**

To address the Reviewer’s comments on the evaluation made in the manuscript we conducted additional analysis and validation carried out for methylation data modality on several datasets, and improved and fixed validation for differential expression analysis. Now we feel that the argument and claims we make regarding the H-score are compelling and plausible.
